# The relationship between perfectionism and symptoms of depression in medical school applicants

**DOI:** 10.1186/s12909-019-1823-4

**Published:** 2019-10-15

**Authors:** Lisa Bußenius, Sigrid Harendza

**Affiliations:** 1 Department of Biochemistry and Molecular Cell Biology, Center for Experimental Medicine, University Medical Center Hamburg-Eppendorf, Hamburg, Germany; 2 III. Department of Internal Medicine, University Medical Center Hamburg-Eppendorf, Hamburg, Germany

**Keywords:** Depression, Medical school admission, Multiple mini-interviews, Perfectionism, Selection for medical school, Undergraduate medical education

## Abstract

**Background:**

The association between perfectionism and depression in the medical profession can ultimately influence physicians’ performance negatively. In medical students, especially maladaptive perfectionism is connected with distress and lower academic performance. The expression of perfectionism and symptoms of depression at the time of medical school application is not known. Therefore, we explored perfectionism and symptoms of depression in participants of multiple mini-interviews for medical school admission and investigated possible differences between applicants who were eventually admitted or rejected.

**Methods:**

After the multiple mini-interviews admission procedure at Hamburg Medical School in August 2018, 146 applicants filled out a questionnaire including sociodemographic data and the following validated instruments: Multidimensional Perfectionism Scale by Hewitt and Flett (MPS-H), Multidimensional Perfectionism Scale by Frost (MPS-F), Patient Health Questionnaire (PHQ-9), Generalized Anxiety Disorder Scale (GAD-7), and a 10-item version of the Big Five Inventory (BFI-10). The two groups of admitted and rejected applicants were compared and the correlation between symptoms of depression and perfectionism further explored.

**Results:**

The admitted applicants were significantly more extrovert and had lower depression scores compared to the rejected applicants. In both groups, the composite scales of Adaptive Perfectionism (*r* = .21, *p* = .011) and Maladaptive Perfectionism (*r* = .43, *p* < .001) as well as their components correlated significantly with the PHQ-9 results. Maladaptive Perfectionism accounted for about 18% of variance in the PHQ-9 score.

**Conclusions:**

Rejected medical school applicants who participated in a multiple mini-interviews admission procedure showed higher levels of depression symptoms than admitted applicants. The degree of depressive symptoms can be partly explained by Maladaptive Perfectionism scores. Since coping in medical school and in postgraduate medical education require robust mental health, perfectionism questionnaires could be an additional tool in medical school selection processes.

## Background

A functioning health care system is of immense importance to society. Yet optimal patient care can be compromised when physicians are unwell [1], suffering from psychopathologies such as depression, anxiety, stress, or burnout [2–5]. A higher rate of medical errors can occur and interpersonal skills such as empathy are affected as well [5]. Students already perceive medical school as a stressful environment, which impedes the expression of empathy [6]. Significant levels of stress and depressive symptoms with an overall prevalence of depression of 27% have been found in medical students [7], which exceeds the depression rates of other students [8, 9] as well as the general population’s [10]. Furthermore, high expression of perfectionism corresponds with higher levels of depressive symptoms and anxiety in medical students [11]. A relationship between symptoms of depression and perfectionism has also been identified in other groups [12–14].

It has been suggested that healthy forms of perfectionism also exist, relating to factors such as conscientiousness, adaptive coping, or positive affect [15]. For example, the setting of high personal standards, a behavior which cannot be considered problematic or pathological per se, is immanent in definitions of perfectionism [16]. The concept of perfectionism has evolved as multidimensional personality characteristics, acknowledging different forms of perfectionism and their effects. One of the most prominent models is the Multidimensional Perfectionism Scale postulated by Frost and colleagues in 1990 [16], which differentiates between Personal Standards, Organization, Concern over Mistakes, and Doubts about Actions on the one hand, and Parental Expectations and Parental Criticism on the other. Another model of perfectionism was established at the same time by Hewitt and Flett, comprising three scales: Self-Oriented Perfectionism, Socially Prescribed Perfectionism, and Other-Oriented Perfectionism [17]. Comparing the two multidimensional perfectionism scales, a factor analysis resulted in two factors, which the authors called maladaptive evaluation concerns and positive achievement strivings [18]. In another study, both Multidimensional Perfectionism Scales were aggregated to form the scales of Adaptive and Maladaptive Perfectionism [19]. The authors found maladaptive perfectionism to correlate with neuroticism and to predict symptoms of distress and dissatisfaction with academic performance in medical students [19].

Many medical schools perform admission tests to select medical school applicants having regard to non-cognitive attributes or psychosocial competencies [20–22]. Multiple mini-interviews (MMIs) have frequently replaced the conventional admission interview [22]. Personality factors like extraversion, conscientiousness, and agreeableness showed weak but reliable correlations to MMI scores [23]. Furthermore, higher levels of extraversion and agreeableness were related to medical school acceptance offers [24]. In Germany, 20% of all available places to study medicine are awarded to high school graduates with the highest grade point averages (GPA) and 20% are reserved to applicants on a waiting list. The remaining 60% are appointed to applicants with a high school degree by selection procedures individually designed by every medical faculty. In 2010, Hamburg Medical School decided to introduce a two-step selection process. While every applicant has to perform a natural science test (HAM-Nat), only the 100 best applicants (about 30%) get direct access to undergraduate medical studies. The applicants with rank 101 to 300 are invited in a second step to participate in a validated MMI called HAM-Int (Hamburg Assessment Test for Medicine – Interview), which assesses the applicants’ psychosocial competencies including empathy, communication skills, and self-regulation [25, 26] and awards the remaining 30% of study places to the approximately 100 best participants.

It is not known how medical school applicants who participate in MMIs score on perfectionism and depression scales. Since both aspects have been shown to have an impact on studying, the aim of our study was to evaluate medical school applicants’ scores for perfectionism and symptoms of depression to elucidate whether differences in these dimensions can be identified between applicants who are accepted for medical school entry and applicants who fail to gain entry to medical school. We further postulate that maladaptive perfectionism can predict the extent of symptoms of depression in medical applicants.

## Methods

### Study design and participants

The enrolment of our study participants took place during the course of the MMI selection process HAM-Int at the Medical Faculty of Hamburg in August 2018. Two hundred applicants were preselected by their high school grade point average (GPA) and a natural sciences test (HAM-Nat). All 194 HAM-Int participants were randomly divided into a morning and an afternoon group and debriefed after finishing the MMI procedure (morning group about 1 pm, afternoon group about 4 pm). In the debriefing phase, participants were asked to participate in this study on a voluntary basis. After informed consent was obtained, the questionnaire was administered to the participants and it took about 15 min to be completed. It contained a barcode identifier and applicants were asked to state their individual admission identification numbers. A key list stored in a separate location was created to enable consolidation of relevant admission data (e.g. HAM-Nat, HAM-Int, and GPA scores with 1 being the best grade) as well as basic sociodemographic aspects (gender and age) by a third party. The questionnaire contained the following validated instruments: Multidimensional Perfectionism Scale by Hewitt and Flett (MPS-H) [27], Multidimensional Perfectionism Scale by Frost (MPS-F) [16], Patient Health Questionnaire (PHQ-9) [28], Generalized Anxiety Disorder Scale (GAD-7) [29], and a 10-item version of the Big Five Inventory (BFI-10) [30]. Applicants signed informed consent prior to the admission procedure and the Ethics Committee of the Hamburg Chamber of Physicians approved of this study (WF-047/16) under the condition that data were anonymized. Therefore, no individual feedback about results to the participants was possible. It was clearly stated that completion of the questionnaire did not impact the HAM-Int score. When the questionnaire was administered, the final admission decision was still unknown to the participants and the authors.

### Instruments

#### Multidimensional perfectionism scale (MPS-H)

The MPS-H includes three scales: Self-oriented perfectionism (SOP), Other-Oriented Perfectionism (OOP) and Socially Prescribed Perfectionism (SPP), each containing 15 items. Items are rated on a 7-point Likert scale (1 indicating disagreement, 7 agreement) [27] (German version by Seeliger and Harendza [31] based on Stoeber [32]). The scales showed satisfactory internal consistency (Cronbach’s alpha = .88, .90 and .72 respectively).

#### Multidimensional perfectionism scale (MPS-F)

This instruments measures six dimensions: Personal Standards (PS) containing seven items, Organization (O) containing six items, Concern over Mistakes (CM) containing nine items, Doubts about Actions (DA) containing four items, Parental Expectations (PE) containing five items and Parental Criticism (PC) containing four items. All 35 items are rated on a 6-point Likert scale (1 being not at all true, 6 being exactly true) [16] (German version by Altstötter-Gleich and Bergemann [33]). Five scales showed acceptable to good internal consistency (O = .93, CM = .86, PE = .85, PC = .77, and PS = .74), while the reliability of the scale DA only showed an internal consistency of .58.

#### Patient health questionnaire (PHQ-9)

This short instrument is used to screen for self-reported symptoms of major depressive disorder (MDD), which makes it a useful research tool [28]. Nine items describing typical depressive states such as having “little interest or pleasure in doing things” are rated on a 4-point Likert scale depending on their occurrence of impairing the participant in the last 2 weeks (0 = not at all, 1 = several days, 2 = more than half the days, 3 = nearly every day). A score ≤ 4 indicates minimal or no signs of MDD, 5–9 indicates a mild form, 10–14 show a moderate severity, 15–19 can be characterized as moderately severe and ≥ 20 is considered a severe form of MDD. To assess global impairment, participants were asked the following question: ‘If you checked off any problems, how difficult have these problems made it to work, take care of things at home, or get along with other people?’ (0 = not at all, 1 = somewhat difficult, 2 = very difficult, 3 = extremely difficult) [28] (German translation by Löwe et al. [34]). The instrument showed good internal consistency (Cronbach’s alpha = .81).

#### Generalized anxiety disorder scale (GAD-7)

In an analog way to PHQ-9, participants were asked about their anxiety level to screen for generalized anxiety disorder (GAD). The scale consists of seven items such as not being able to stop or control worrying. The same Likert scale applies. A score ≤ 4 indicates minimal or no signs of GAD, 5–9 indicates a mild form, 10–14 show a moderate severity while ≥15 can be considered a severe form of GAD [29] (German translation by Löwe et al. [35]). This instrument also showed good reliability (Cronbach’s alpha = .88).

#### Big five inventory, 10-item version (BFI-10)

On this short version of the BFI, which was used in our previous study [31], five personality dimensions including Openness, Conscientiousness, Extraversion, Agreeableness, and Neuroticism are measured by means of two items each on a 5-point Likert scale (1 = disagree strongly, 5 = agree strongly; both German and English version by Rammstedt and John [30]). The Cronbach’s alpha values were: Openness = .69, Extraversion = .63, Conscientiousness = .61, Neuroticism = .53, and Agreeableness = .30.

### Data processing

The data were obtained from the questionnaires by means of optical mark recognition (Remark Office OMR, pki Informationssysteme für Marktforschung, Hamburg, Germany) and spot-checked for errors. The data were analyzed using IBM SPSS Statistics for Windows, version 25 (IBM Corp., Armonk, N.Y., USA) with a general alpha-level of .05. The applicants’ final ranking is based on the sum of GPA, HAM-Nat, and HAM-Int scores. The scores are transformed onto a linear scale so that the GPA accounts for 34% of the final score, while HAM-Nat and HAM-Int account for 33%, respectively. The GPA in Germany is measured on a scale from 1 (best grade) to 6 (worst grade), and is transformed onto a linear scale from 0 to 60 (60 being the highest score). HAM-Nat and HAM-Int scores are both transformed onto a linear scale from 0 to 59 (59 being the highest score). The instruments obtained from the questionnaire were included in the data set when at least 80% of the items were rated. Missing data were handled through imputation of the mean of the scale. The composite measure of Adaptive Perfectionism (AP) was calculated using the *z*-transformed scores of the subscales SOP and PS. Maladaptive Perfectionism (MP) was calculated using the *z*-transformed subscales SPP, CM, and DA. Group differences between admitted and rejected applicants were calculated with independent t-tests or Mann-Whitney-U-tests for ordinal scaled data. Where applicable, Cohen’s *d* was calculated to estimate effect size. The general association of the subscales of perfectionism with symptoms of depression was assessed using Pearson correlations. The *z*-standardized scale of Maladaptive Perfectionism was included as predictor in a linear regression model for PHQ-9 to test the hypothesis that maladaptive forms of perfectionism can predict symptoms of depression.

## Results

Of the 194 applicants who took part in the MMI selection procedure, 152 (78%) participated voluntarily in our study. Six questionnaires had to be excluded before data processing due to lack of consent to data consolidation. No other exclusion criteria where applicable. Thus, the final sample consisted of 146 applicants (94 female, 64%). Mean age was 19.8 ± 1.63 years, with a minimum age of 17 and a maximum age of 27 years. Mean GPA was 1.52 ± 0.16, mean HAM-Nat score was 37.91 ± 3.75, and mean HAM-Int score was 35.37 ± 4.76. Mean total score, on which the applicants’ final ranking was based, was 122.84 ± 5.12, consisting of GPA (34%), HAM-Nat result (33%), and HAM-Int result (33%). Of the 146 applicants, 92 were eventually admitted according to the acceptance quota, while 54 were rejected. Participants’ characteristics can be obtained from Table 1. The groups did not differ significantly in age and gender. While there was no significant difference in their HAM-Nat score, the admitted applicants showed significantly higher GPAs, *t* (144) = − 2.02, *p* = .046 as well as significantly higher HAM-Int scores, *t* (114) = 12.29, *p* < .001. As the final admission decision is based on the ranking by each applicant’s total score consisting of GPA, HAM-Nat and HAM-Int scores, the group of admitted applicants showed significantly higher total scores, *t* (114) = 14.56, *p* < .001.

**Table 1:** Participants’ characteristics

	Applicant sample	Admitted applicants	Rejected applicants	Cohen’s d
Sociodemographic data				
N	146	92	54	
Sex (female/male)	94/52	62/30	32/22	
Age (mean ± SD)	19.85 ± 1.63	19.79 ± 1.78	19.94 ± 1.35	0.09
Selection criteria (mean ± SD)				
GPA	1.52 ± 0.16	1.50 ± 0.16	1.56 ± 0.16*	0.38
GPA score	49.56 ± 3.29	49.98 ± 3.30	48.85 ± 3.19*	0.35
HAM-Nat score	37.91 ± 3.75	37.91 ± 3.88	37.91 ± 3.55	0.00
HAM-Int score	35.37 ± 4.76	37.97 ± 3.20	30.94 ± 3.57***	2.10
Total score	122.84 ± 5.12	125.86 ± 3.06	117.70 ± 3.60***	2.50
Personality measures (mean ± SD)				
Patient Health Questionnaire	7.19 ± 4.70	6.57 ± 4.56	8.28 ± 4.81*	0.37
Generalized Anxiety Disorder Scale	6.85 ± 4.83	6.56 ± 5.03	7.35 ± 4.47	0.16
Big Five Inventory, 10 item version				
Openness	7.35 ± 2.14	7.41 ± 2.10	7.26 ± 2.21	0.07
Conscientiousness	7.89 ± 1.64	8.04 ± 1.54	7.62 ± 1.79	0.26
Extraversion	7.10 ± 1.82	7.38 ± 1.66	6.62 ± 1.99*	0.43
Agreeableness	7.29 ± 1.61	7.48 ± 1.49	6.94 ± 1.76	0.34
Neuroticism	5.14 ± 1.75	5.03 ± 1.66	5.32 ± 1.88	0.17

*: *p* < .05, ***: *p* < .001

Regarding the personality measures included in the questionnaire, the rejected applicants showed a significantly higher score in the PHQ-9 with a mean of 8.28 than the admitted applicants with a mean of 6.57, *t* (143) = − 2.14, *p* = .034. Following Kroenke and Spitzer’s recommendation of classification of PHQ-9 scores [28], over 25% of all participants showed a clinically relevant score of ≥10, indicating moderate symptoms of depression levels or higher. Analysis of the item for global impairment in the PHQ-9 showed a significant difference between admitted and rejected applicants, *U* = 1647.5, *p* = .010, indicating a higher level of impairment in rejected applicants (*Mdn* = 2) than admitted applicants (*Mdn* = 1). The applicants showed no significant differences in their general anxiety scores in the GAD-7. Regarding the 10 item version of the BFI, admitted and rejected applicants showed no differences in the four domains Openness, Conscientiousness, Agreeableness, or Neuroticism, but the admitted applicants were significantly more extrovert than the rejected applicants, *t* (143) = 2.46, *p* = .015 (Table 1). Comparing the subscales of the perfectionism measures, MPS-H and MPS-F did not result in significant differences between the two groups. While the strongest correlation with PHQ-9 was found for Maladaptive Perfectionism (*r* = .43, *p* < .001), Self-Oriented Perfectionism (*r* = .26, *p* = .002), Socially Prescribed Perfectionism (*r* = .38, *p* < .001), Concern over Mistakes (*r* = .37, *p* < .001), and Doubts about Actions (*r* = .34, *p* < .001) all correlated moderately with the PHQ-9 score as did Adaptive Perfectionism to a lesser extent (*r* = .21, *p* = .011, Table 2).

**Table 2:** Group differences in the dimensions of perfectionism and correlations with depression scores

	Admitted applicants	Rejected applicants	*r*_*PHQ-9*_
MPS-H			
Self-Oriented Perfectionism	71.22 ± 14.76	72.57 ± 14.49	.26**
Socially Prescribed Perfectionism	43.17 ± 15.23	45.49 ± 13.60	.38***
Other-Oriented Perfectionism	49.10 ± 10.24	48.62 ± 9.67	.19*
MPS-F			
Personal Standards	30.17 ± 4.99	30.31 ± 4.62	.12
Organization	27.82 ± 6.44	27.45 ± 6.40	.07
Concern over Mistakes	20.86 ± 7.82	21.96 ± 7.64	.37***
Doubts about Actions	11.14 ± 3.53	11.70 ± 3.58	.34***
Parental Expectations	11.80 ± 5.17	12.49 ± 5.46	.15
Parental Criticism	7.04 ± 3.71	7.57 ± 3.05	.13
Composite scales (*z*-standardized)			
Adaptive Perfectionism	-0.04 ± 1.86	0.08 ± 1.71	.21*
Maladaptive Perfectionism	-0.17 ± 2.68	0.28 ± 2.30	.43***

*MPS-H* Multidimensional perfectionism scale by Hewitt & Flett, *MPS-F* Multidimensional perfectionism scale by Frost

*: *p* < .05, **: *p* < .01, ***: *p* < .001

The differences in depressive symptom levels between the two groups were further explored with a linear regression model including Maladaptive Perfectionism. All three independently assessed models showed a significant explanatory power of Maladaptive Perfectionism to predict symptoms of depression to a similar extent. The predictor accounts for 18% of the variance in PHQ-9 scores in the total sample of applicants, and slightly more in rejected applicants (20%) than in admitted applicants (17%, Table 3).

**Table 3:** Linear regression analysis for prediction of symptoms of depression by Maladaptive Perfectionism

Model	N	*F*	df	*R* ^*2*^	stand. β	*T*	*p*
PHQ-9 of total sample	146	31.73	1143	.18	.43	5.63	< .001
PHQ-9 of admitted applicants	92	18.28	1,90	.17	.41	4.28	< .001
PHQ-9 of rejected applicants	54	12.32	1,51	.20	.44	3.51	.001

*PHQ-9* Patient Health Questionnaire, *stand.* standardized

## Discussion

In our study, rejected applicants showed significantly higher scores for symptoms of depression than admitted applicants and felt significantly more affected by depressive symptoms in daily life. They stated that the depressive symptoms made it “very difficult to work, take care of things at home, or get along with other people”. Since depressive mood can predict lower performance in situations of high personal importance [36, 37], the elevated global impairment in our rejected applicants might reflect their lower performance levels in the multi mini-interview. Additionally, admitted applicants in our study showed significantly higher scores for extraversion in the BFI-10 than rejected applicants. This is in line with the literature stating extraversion to be associated with better MMI performance [23, 24]. Extraversion is a social trait which is required to cope with the demands of undergraduate medical education and when faced with unfamiliar medical situations or new patients during postgraduate medical practice [38]. Furthermore, a meta-analysis on links between perfectionism and personality traits revealed that perfectionistic concerns defining Maladaptive Perfectionism were associated with lower levels of extraversion [39].

Maladaptive Perfectionism accounted for about 18% of variance in the PHQ-9 scores of our medical school applicants. This finding is in line with our hypothesis that Maladaptive Perfectionism can predict symptoms of depression in medical applicants. Maladaptive Perfectionism comprises Socially-Prescribed Perfectionism (the belief that others place high standards on oneself), Concern over Mistakes (negative reactions to own mistakes), and Doubts about Actions (the belief that performance can never lead to satisfaction), all reflecting maladaptive evaluative concerns and impeding performance [19]. Furthermore, it has been shown that perfectionistic attitudes in general are an attendant circumstance of depressive symptoms and might in turn mediate the relationship between depression and stress [14]. Hence, these aspects of perfectionism seem to be undesirable in medical school applicants. Some students who are admitted to medical school show high levels of psychological distress at the time of admission and remain at these high levels while they progress through medical school [40]. While most medical students’ emotional state is comparable to that of the general population, for some individuals depressive symptoms scores increase and persist over time [8]. Therefore, medical schools need to acknowledge the increase in depressive symptoms during undergraduate medical education [41] and promote resilience in their medical students [42], which could be achieved, for instance, by offering creative programs and courses about managing a stressful environment [43]. Resilience strategies, e.g. gratification from medical efficacy as well as from the doctor-patient relationship, self-demarcation and self-awareness [44] need to be fostered, which are required to maintain the physician’s behavior patients need [45]. Because robust mental health is required for the demands of physicians’ daily practice, integration of such aspects into selection procedures for medical school has been a matter of debate [46]. With respect to our findings we propose to assess aspects of perfectionism rather than clinical symptoms in addition to other medical school admission processes like multiple mini-interviews. Whether perfectionism questionnaires can also be used in selection procedures to assess Adaptive Perfectionism, where high levels would be highly desirable in medical students because Adaptive Perfectionism can be associated with positive outcomes such as adaptive coping or positive affect [15], but unfortunately decreases during undergraduate medical training [47], needs to be further investigated.

One weakness of our study is that the admitted and rejected groups not only differ in certain aspects of perfectionism and in the extent of showing symptoms of depression but also in some socio-demographic aspects. Admitted applicants had higher GPAs and were also significantly more extroverted. The admission quota after the HAM-Int process varies annually according to capacity, thus the two groups – admitted and rejected students – do not show equal sample sizes. Furthermore, we cannot draw conclusions with regard to the big five personality traits, as the BFI-10 showed rather poor reliability scores and might even underrepresent the constructs. We used this short version for reasons of feasibility, but a more extensive instrument needs to be employed for results with higher reliability. Another weakness of our study is that it remains uncertain whether our findings can be generalized, as this study only took place at one medical school and entry regulations differ immensely between schools and countries. Furthermore, we will not be able to demonstrate that our sample is representative for medical school applicants, since every summer about 43,000 people apply for medical studies in Germany and we cannot show the sociodemographic data of these applicants nor will we be able to find out who was admitted to a medical school. Over 25% of all participants in our study showed a clinically relevant score indicating moderate symptoms of depression levels or higher. This number seems very high compared to the prevalence of depressive symptoms in the general German population [48] and in another population of German medical students at the beginning of their first semester [31]. The time point of administration of the questionnaire in our study – after the participation in the multiple mini-interview – could have played a relevant role for this result, because the timeframe that has to be evaluated in the PHQ-9 – the past fortnight – coincides with the invitation to and preparation for the multiple mini-interviews. Furthermore, participants of the morning group showed significantly (*p* = .045) higher scores in Conscientiousness (Additional file 1: Table S1). This might be due to an incidental overrepresentation of females in the morning group (62 females and 17 males) versus the afternoon group (32 females and 35 males), because females showed significantly (*p* = .017) higher scores for Conscientiousness than males. However, admitted applicants still had significantly lower scores in the PHQ-9 than rejected applicants. They might have been affected by their subjective feelings of their performance when completing the questionnaire, which could have led to a bias in the answers. A strength of our study is the return rate of 78% which provides a good quota of the total sample. Even though the effect sizes of the significant differences in the perfectionism scores between the admitted and rejected applicants are small, testing for aspects of perfectionism might be an interesting addition in the selection process for medical school to reduce the number of admitted students who are at greater risk to develop symptoms of depression.

## Conclusions

Medical school applicants who were eventually rejected after a multiple mini-interview selection procedure showed significantly higher levels of symptoms of depression than admitted applicants. In general, the extent of depressive symptoms could be partly explained by Maladaptive Perfectionism. Since Maladaptive Perfectionism hampers performance during undergraduate medical education as well as postgraduate education, assessing the different levels of perfectionism could be additionally recommended for medical school selection to admit those with lower risk of developing symptoms of depression.

## Supplementary information

**Additional file 1: Table S1.** Participants’ characteristics according to the time points of data collection.

## Data Availability

All data and materials are available from the corresponding author upon request.

## Abbreviations

AP: Adaptive perfectionism
BFI-10: Big Five Inventory 10
CM: Concern over Mistakes
DA: Doubts about Actions
GAD: Generalized anxiety disorder
GAD-7: Generalized Anxiety Disorder Scale
GPA: High school grade point average
HAM-Int: Hamburg Assessment Test for Medicine – Interview
MDD: Major depressive disorder
MMI: Multiple mini-interview
MP: Maladaptive Perfectionism
MPS-F: Multidimensional Perfectionism Scale by Frost
MPS-H: Multidimensional Perfectionism Scale by Hewitt and Flett
O: Organization
OOP: Other-Oriented Perfectionism
PC: Parental Criticism
PE: Parental Expectations
PHQ-9: Patient Health Questionnaire
PS: Personal Standards
SOP: Self-Oriented Perfectionism
SPP: Socially-Prescribed Perfectionism
