# Supplementary material for: The relationship between perfectionism and symptoms of depression in medical school applicants
Source: BMC Med Educ. 2019 Oct 15;19:370. doi: 10.1186/s12909-019-1823-4 (PMC6794862; doi:10.1186/s12909-019-1823-4)
Supplement: Supplementary file 1 — Additional file 1: Table S1. Participants’ characteristics according to the time points of data collection. [file 12909_2019_1823_MOESM1_ESM.docx]

**Table S1: Participants’ characteristics according to the time points of data collection**

|  | **Applicant sample** | | **Morning group** | | **Afternoon group** | | **Cohen’s d** |
| --- | --- | --- | --- | --- | --- | --- | --- |
| **Sociodemographic data** | | | | | | | |
| N | 146 | | 79 | | 67 | |  |
| Sex (female/male) | 94/52 | | 62/17 | | 32/35*** | | 0.32^¶^ |
| Age (mean ± SD) | 19.85 | ± 1.63 | 19.96 | ± 1.60 | 19.72 | ± 1.68 | 0.15 |
| **Selection criteria** (mean ± SD) | | | | | | | |
| GPA | 1.52 | ± 0.16 | 1.51 | ± 0.14 | 1.54 | ± 0.18 | 0.15 |
| GPA score | 49.56 | ± 3.29 | 49.80 | ± 2.82 | 49.28 | ± 3.78 | 0.15 |
| HAM-Nat score | 37.91 | ± 3.75 | 37.64 | ± 3.40 | 38.23 | ± 4.13 | 0.16 |
| HAM-Int score | 35.37 | ± 4.76 | 35.86 | ± 4.89 | 34.80 | ± 4.57 | 0.22 |
| Total score | 122.84 | ± 5.12 | 123.30 | ± 5.18 | 122.31 | ± 5.05 | 0.19 |
| **Multidimensional Perfectionism Scale by Hewitt & Flett** (mean ± SD) | | | | | | | |
| Self-Oriented Perfectionism | 71.72 | ± 14.62 | 71.61 | ± 14.96 | 71.84 | ± 14.32 | 0.02 |
| Socially Prescribed Perfectionism | 44.03 | ± 14.64 | 43.78 | ± 15.00 | 44.32 | ± 14.31 | 0.04 |
| Other-Oriented Perfectionism | 48.92 | ± 10.00 | 48.71 | ± 9.92 | 49.17 | ± 10.17 | 0.05 |
| **Multidimensional Perfectionism Scale by Frost** (mean ± SD) | | | | | | | |
| Personal Standards | 30.22 | ± 4.84 | 30.14 | ± 4.70 | 30.32 | ± 5.04 | 0.04 |
| Organization | 27.68 | ± 6.40 | 28.53 | ± 6.32 | 26.66 | ± 6.39 | 0.29 |
| Concern over Mistakes | 21.26 | ± 7.75 | 20.56 | ± 7.75 | 22.11 | ± 7.73 | 0.20 |
| Doubts about Actions | 11.34 | ± 3.55 | 11.14 | ± 3.65 | 11.59 | ± 3.43 | 0.13 |
| Parental Expectations | 12.06 | ± 5.27 | 11.54 | ± 5.00 | 12.66 | ± 5.55 | 0.21 |
| Parental Criticism | 7.24 | ± 3.48 | 7.20 | ± 3.66 | 7.28 | ± 3.28 | 0.02 |
| **Composite scales (*z*-standardized)** | | | | | | | |
| Adaptive Perfectionism | 0 | ± 1 | -0.02 | ± 1.83 | 0.03 | ± 1.79 | 0.03 |
| Maladaptive Perfectionism | 0 | ± 1 | -0.17 | ± 2.61 | 0.19 | ± 2.49 | 0.14 |
| **Personality measures** (mean ± SD) | | | | | | | |
| Patient Health Questionnaire | 7.19 | ± 4.70 | 6.96 | ± 4.77 | 7.47 | ± 4.65 | 0.11 |
| Generalized Anxiety Disorder  Scale | 6.85 | ± 4.83 | 6.52 | ± 5.14 | 7.24 | ± 4.44 | 0.15 |
| Big Five Inventory, 10 item version | |  |  |  |  |  |  |
| Openness | 7.35 | ± 2.14 | 7.65 | ± 1.91 | 7.02 | ± 2.37 | 0.29 |
| Conscientiousness | 7.89 | ± 1.64 | 8.14 | ± 1.62 | 7.59 | ± 1.64* | 0.34 |
| Extraversion | 7.10 | ± 1.82 | 7.08 | ± 1.81 | 7.14 | ± 1.85 | 0.03 |
| Agreeableness | 7.29 | ± 1.61 | 7.51 | ± 1.48 | 7.02 | ± 1.73 | 0.31 |
| Neuroticism | 5.14 | ± 1.75 | 5.24 | ± 1.77 | 5.02 | ± 1.71 | 0.13 |

Notes: Time points of data collections are 1 pm for the morning group and 4 pm for the afternoon group. *: p < .05, **: p < .01, ***: p < .001. ^¶^The effect size applicable for the χ^2^-test used here is Phi ɸ.
